# Experimental and Numerical Investigations on the Thermomechanical Behavior of 304 Stainless Steel/Q345R Composite Plate Weld Joint

**DOI:** 10.3390/ma12213489

**Published:** 2019-10-24

**Authors:** Xiaodong Hu, Yicheng Yang, Ming Song

**Affiliations:** 1 College of Mechanical and Electronic Engineering, Shandong University of Science and Technology, Qingdao 266590, China; huxdd@163.com (X.H.);; 2 College of Pipeline and Civil Engineering, China University of Petroleum (East China), Qingdao 266580, China

**Keywords:** composite plate, thermomechanical behavior, microstructure, finite element analysis, welding residual stress, element diffusion

## Abstract

In this study, the welding process of 304 stainless steel/Q345R low alloy steel composite plate is modeled by experimental and finite element methods to study the complex thermomechanical behavior. The residual stress and microstructure evolution of composite plate in the welding process are also investigated. The welding thermal cycle curve and residual stress distribution at the joint are obtained by using thermocouple and blind-hole methods. Optical microscopy, scanning electron microscopy, and energy-dispersive X-ray spectroscopy were used to investigate the evolution of microstructure, morphology, and element diffusion of the joint. The results show that the maximum von Mises welding residual stress is 312 MPa, which is located in the bottom of the start point of the weld zone. The residual stress gradually decreases and tends to be stable along the direction from the weld to the base metal. In addition, a residual stress discontinuity is found at the interface between the bimetal. It is also found that the closer it is to the weld joint, the more uniform is the austenite distribution and the smaller are the grain sizes.

## 1. Introduction

Austenitic stainless steel (SS)/low alloy steel composite plates have been widely used in petroleum and chemical industries due to their good weldability and formability. The 304 SS/Q345R composite plate is composed of Q345R low alloy steel as the base material and 304 SS is laminated on the surface by using explosive welding [1]. It not only has good welding performance and high strength coming from the low alloy steel, but also has the excellent characteristics of corrosion resistance, wear resistance, and high thermal conductivity coming from the stainless steel [2]. However, there are many problems, related to the thermomechanical behavior, which are not completely understood and unsolved in the welding and repair welding of the composite plate due to the complex structure of the bimetal composite. So far, many studies on numerical simulation of the welding process have been conducted. Deng et al. [3] developed a thermo-elastic-plastic finite element method to calculate the residual stress and welding deformation of 304 SS butt joint with a thickness of 10 mm in various groove forms. Also, many studies have proved that using the finite element method to analyze the mechanical behavior of the welding was reliable. Jiang et al. [4,5] evaluated the residual stress distribution through thickness in the welding process of 304 SS composite plate by using neutron diffraction and finite element analysis (FEA). Ding et al. [6] studied the mechanical properties of the weld joint of 304 SS/Q345R composite plate and found that the hardness of the clad layer increased significantly in the welding heat affected zone (HAZ) and decreased significantly in the weld zone. The hardness of the base layer increased slightly in the HAZ. However, due to the complex structure of the bimetal composite plate, the relationship between the thermomechanical behavior and microstructure evolution during the welding process and the distribution of the thermal stress and post-weld residual stress is still not clear. Besides, very few studies have been reported on repair welding of the composite plate. Therefore, the study on the evolution of residual stress and microstructure in the welding and repair welding of a composite plate weld joint is necessary. It is also important to investigate the element diffusion of the interface of the bimetal joint. In the welding process of the composite plate composed of austenitic stainless steel and low alloy steel, the compounds and content of elements in the welding zone and the adjacent HAZ are very different. In the literature [7], SA 508 and 316L SS are welded with ERNiCr-7 filler rod by tungsten inert gas (TIG) welding, and it was found that dendritic structure and compounds of TiC, TiN, and Al_2_MgO_4_ were generated in the welding seam. When 304 SS and SA 553 are welded by high-energy laser, a few ferrites are found to be generated near the fusion zone of 304 SS, and M_23_C_6_ carbides were found in grain boundaries; a small amount of bilayer-structure inclusions was also found in the grain [8].

In this study, a 304 SS/Q345R composite plate weld joint was prepared, and a groove was trenched and repaired by several weldings. The evolution of the microstructure and residual stress distribution of the composite weld joint were studied by experimental and numerical methods based on the thermos-elastic-plastic theory. The Schaeffer phase diagram [9] was used to predict the phase composition of the welding joint. The influence of the welding temperature field evolution on the microstructure and thermomechanical properties of the welding joint was discussed. This study aims to provide a combined experimental and numerical method for the welding and repair welding process design and optimization of SS/low alloy steel composite plates.

## 2. Experimental Procedure

### 2.1. Materials and Welding Process

**Figure 1:**
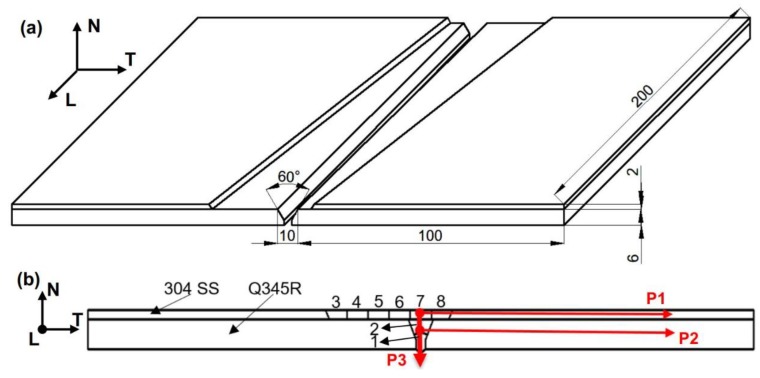
(**a**) Schematic of 304 SS/Q345R composite plate and (**b**) the cross section of welded joint of composite plate. P1, P2, and P3 are the residual stress measurement paths.

Figure 1a shows the diagram of the 304 SS/Q345R composite plate with a groove for welding and a trenched layer for repair welding. *N*, *T*, and *L* represent the normal, transverse, and longitudinal directions, respectively. In order to study the complex thermomechanical behaviors of repair welding of the composite plate and the influence of base welding on the groove, the composite layer was trenched. The two pieces of 304 SS/Q345R composite plates were welded together first and then the trenched layer was filled by several repair welding. The sequence of welding and repair welding is shown in Figure 1b. The dimension of each composite plate is 200 × 100 × 8 mm. The base metal is Q345R with a thickness of 6 mm. The clad metal is 304 SS with a thickness of 2 mm. The trenched layer is an inclined welding bead with a width of 40 mm and is shaved off at the composite layer as shown in Figure 1a. Their chemical compositions are listed in Table 1. Er50-6, J507, and 302 filler materials are selected as welding rods in the experiment, and their chemical compositions are listed in Table 2. Figure 1b also shows the shape of V groove in the base metals with an angle of 60 degrees.

Table 3 shows detailed welding parameters. In order to ensure the mechanical properties and the corrosion resistance of the weld joint [10], the base metal is welded twice in sequence and the clad layer was welded six times. The base layer of low alloy steel is welded in the order of 1 and 2, and the upper SS layers are welded in the order of 3, 4, 5, 6, 7, and 8 as shown in Figure 1b. Q345R base layer and 304 SS clad layer groove were all welded and repair welded by manual arc welding (MAW). In MAW, a direct current (DC) positive electrode connection was adopted to protect the welding seam and improve welding performance. After welding, the strength and hardness of the composite plate are improved, but the ductility is reduced. Therefore, it is generally required to eliminate the residual stress of the composite plate through heat treatment or ultrasonic vibration method. The welding voltage, current, and linear velocity are mainly controlled in the welding process. In the composite welding, a welding procedure with filler rods of small diameter, small current, multi-pass welding, and shallow depth of fusion was adopted to effectively control element dilution between dissimilar metals [11].

### 2.2. Measurement of Welding Heat Cycle Curve

**Figure 2:**
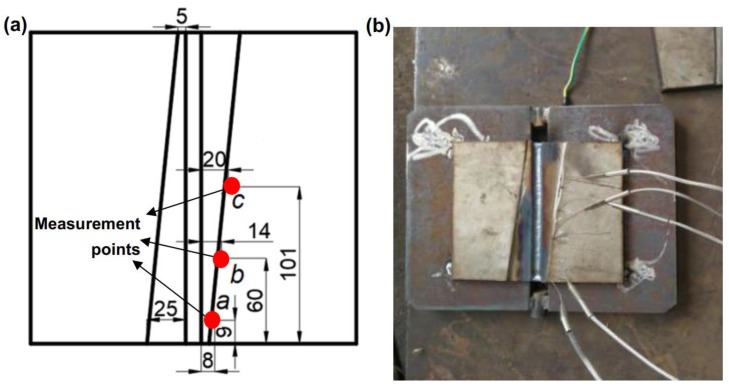
(**a**) Schematic of the points *a*, *b*, and *c* for measuring welding heat cycle curve and (**b**) illustration of thermocouple installation.

Figure 2a shows the specific temperature collection points, which are measured by three thermocouples attached to the plate as shown in Figure 2b. The HPDJ-8125 dynamic data acquisition and analysis system was used to collect the transient temperatures during the welding process. The whole welding process was carried out according to the proposed welding procedure as shown in Table 3, and the corresponding data of temperature evolution were collected. Thus, the welding heat cycle curve of the collection points could be obtained.

### 2.3. Residual Stress Measurement by Blind-Hole Method

**Figure 3:**
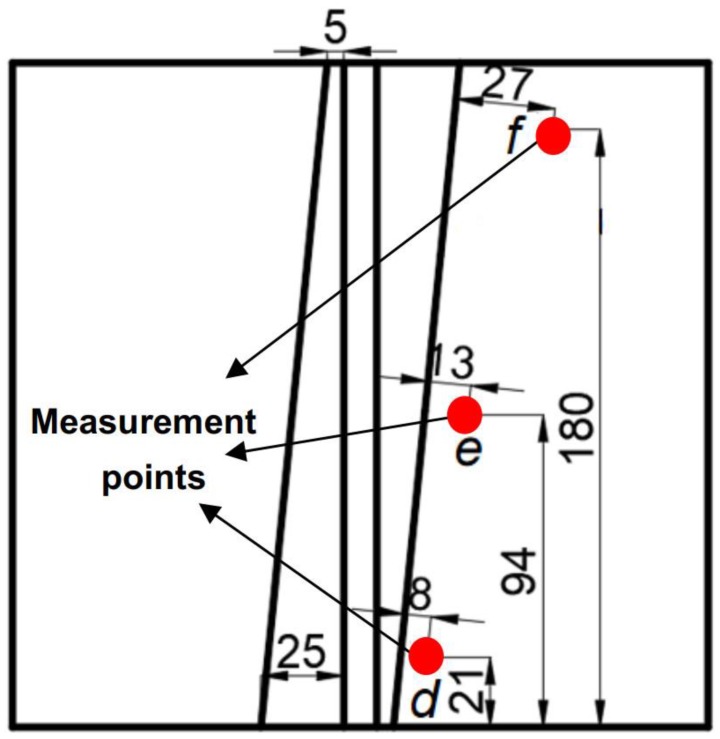
Illustration of the residual stress measurement points *d*, *e*, and *f*.

Figure 3 shows the schematic of residual stress measurement points *d*, *e*, and *f*. The blind-hole method was adopted to measure the residual stress distribution in HAZ. The HT21B portable digital residual stress detector is used to obtain the residual stresses at *d*, *e*, and *f* points. Firstly, an abrasive paper was used to polish the test area until the surface was smooth; secondly, alcohol-soaked cotton balls were used to clean the surface to keep it free of oil and impurities; thirdly, strain gauges were fixed to the test points of the composite plate with glue; and finally, several blind holes with a dimension of 2 mm diameter and 1.5–2 mm depth were drilled at the measurement points. The values of residual stresses were recorded after the hole was finished.

### 2.4. Microstructure Characterization

**Figure 4:**
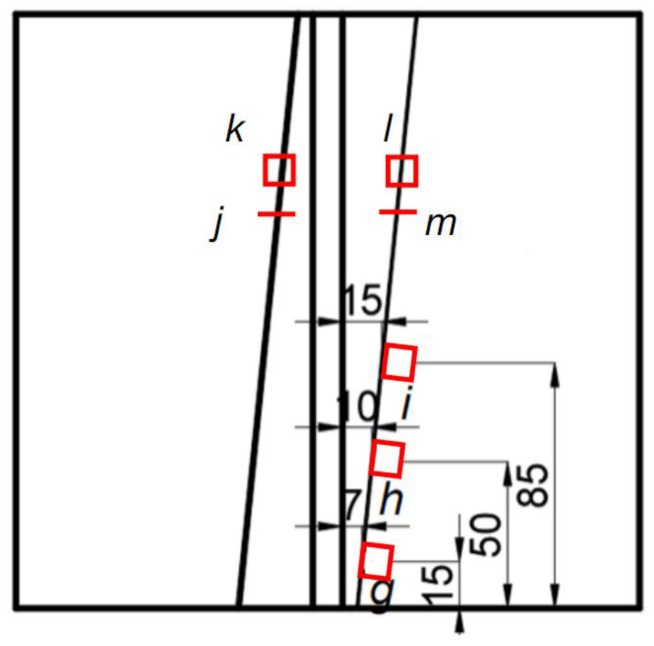
Schematic of specimens *g*, *h*, *i*, *j*, *k*, *l*, and *m* sampled from the composite plate for microstructure characterization, red short lines and red rectangles represent the surface prepared for microstructure characterization.

After welding and residual stress measurement, the sample was machined by electron discharge machining (EDM) method from the weld joint, with a dimension of length, width, and thickness of 10 mm × 10 mm × 8 mm, respectively. Figure 4 shows the specimen sampling positions in the composite layer. The microstructures of the three samples (*g*, *h*, and *i*) were observed. Aqua regia was prepared as an etchant by adding three parts hydrochloric acid (37 wt.% HCl) to one part nitric acid (67 wt.% HNO_3_) to display the microstructure of the weld joint of the base layer and the clad layer [12]. An optical microscope was used to observe the microstructure of the specimens sampled. Energy-dispersive X-ray spectroscopy (EDS) was used to analyze the change in the elemental composition of the spots near the fusion area.

### 2.5. Finite Element Modeling

**Figure 5:**
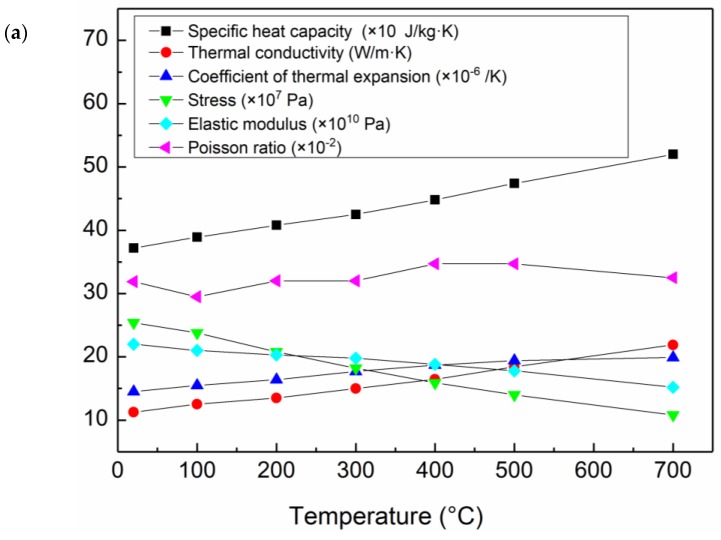

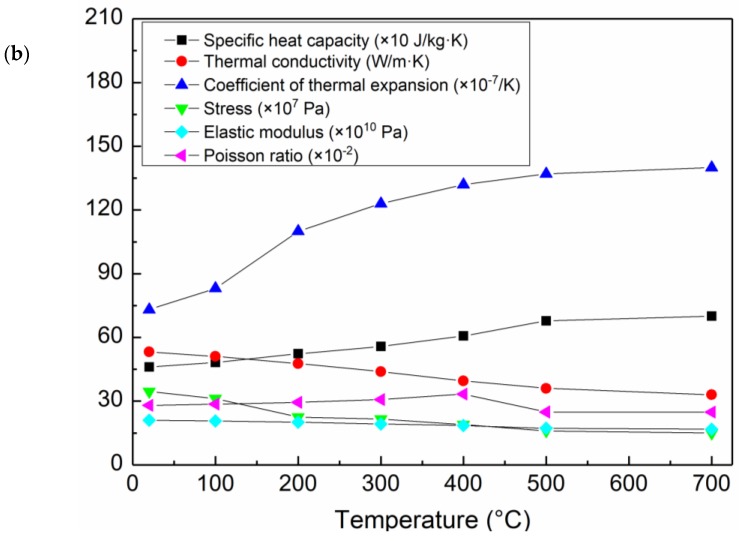
(**a**) Thermophysical properties of 304 SS and (**b**) Q345R low alloy steel used in the finite element model.

Figure 5 shows the thermophysical properties of 304 SS and Q345R. The heat source of the double ellipsoid [13] was adopted in the heat source modeling of the finite element analysis. The volumetric heat source of the double ellipsoid model moving on the plate was divided into an anterior hemisphere and a posterior hemisphere. Analytical heat source expression of the anterior hemisphere *q*_a_ and the posterior hemisphere *q*_p_ is as follows:

(1) $$q_{a}(x,y,z)=\frac{6\sqrt{3}f_{f}q_{0}}{abc_{f}\pi \sqrt{\pi }}exp\left(-\frac{3x^{2}}{c_{f}{}^{2}}-\frac{3y^{2}}{a^{2}}-\frac{3z^{2}}{b^{2}}\right),$$

(2) $$q_{p}(x,y,z)=\frac{6\sqrt{3}f_{b}q_{0}}{abc_{b}\pi \sqrt{\pi }}exp\left(-\frac{3x^{2}}{c_{b}{}^{2}}-\frac{3y^{2}}{a^{2}}-\frac{3z^{2}}{b^{2}}\right),$$

where *a* and *b* are the semiaxes of the ellipsoid *y* and *z* directions, *c*_f_ and *c*_b_ are the semiaxes of the front and rear ellipsoids in the *x* direction, *f*_f_ and *f*_b_ are the energy distribution coefficients in the front and the rear parts, respectively, and satisfy the equation of $f_{f}+f_{b}=2$. *q*_0_ is the power of welding heat input. The movement of the heat source was controlled by changes in welding speed and welding time.

In the welding temperature distribution, all the outer surfaces of the welding plates were selected as convective heat exchange surfaces, and the initial temperature was set to room temperature (RT; 20 °C) before welding. Thermal radiation and thermal convection are the main ways of heat exchange between welding and environment. In the modeling, the emissivity was 0.85, the Stefan-Boltzmann constant was 5.67 W/(m^2^·K^4^), and the convective heat transfer coefficient was 10 W/(m^2^·K). The body heat flux acting on the weld was set as 1. In the simulation of welding residual stress distribution, the displacement of the bottom surface in the *L* and *T* directions was constrained to avoid rigid movement.

**Figure 6:**
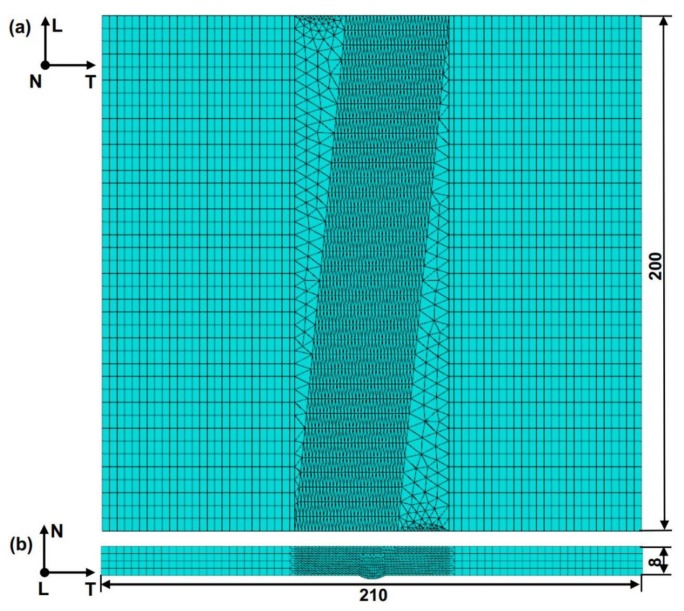
Finite element model and meshing, (**a**) top view and (**b**) cross section.

As shown in Figure 6, the three-dimensional finite element geometry model was mainly divided into two parts: low alloy steel base layer and SS clad layer. The base layer and the clad layer were bonded together. In total, 20,763 nodes and 46,892 elements were meshed, and the element type of the model was created by a combination of C3D4 with C3D8R elements. The C3D4 element is a general purpose tetrahedral element with one integration point and the C3D8R element is a general purpose linear brick element with reduced integration (one integration point). In the process of mesh generation, the mesh size at the weld was refined and the relative larger size mesh was applied in the base metal layer. Between these two mesh zones, a transition mesh zone was applied in the HAZ to link them.

## 3. Results and Discussion

### 3.1. Effects of Temperature Distribution and Evolution

**Figure 7:**
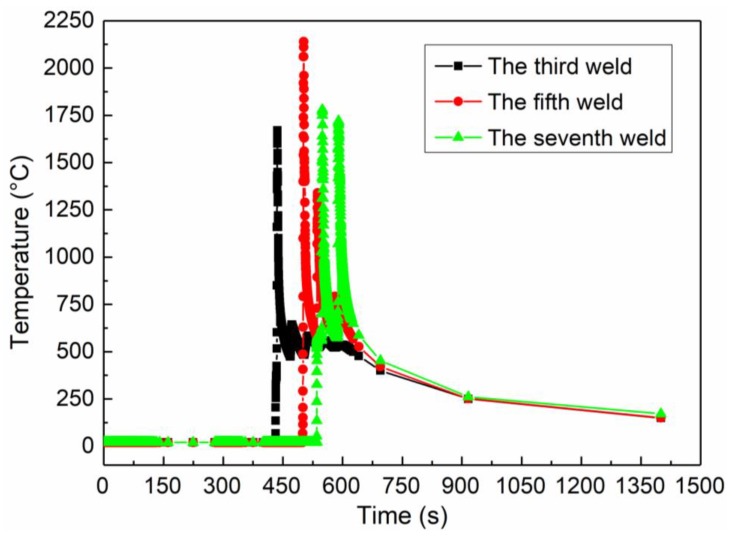
Welding heat cycle curves obtained from finite element analysis.

In order to study the temperature variation of the composite plate during welding in FEA, three nodes were selected to observe the thermal cycle curve, which presents temperature changes with time. The three nodes are located at the center of the third welded clad layer, the center of the fifth weld clad layer, and the center of the seventh weld clad layer. In general, all of the selected nodes are located at the center of the welded clad layers. Figure 7 shows the thermal cycle curve of each node. It can be seen from Figure 7 that the temperature rises sharply when the heat source passes the node. After the heat source passes, with the distance between the heat source and the selected node increasing, the temperature decreases with time. Until another weld bead begins to weld, the temperature increases again. During the welding process, the influence of the heat source on the selected node will gradually decrease with the increase in the distance between the welding joint and the measured point, and the maximum value of each curve will decrease successively with time. The maximum temperature in the welding is around 2140 °C in the third welding process.

**Figure 8:**
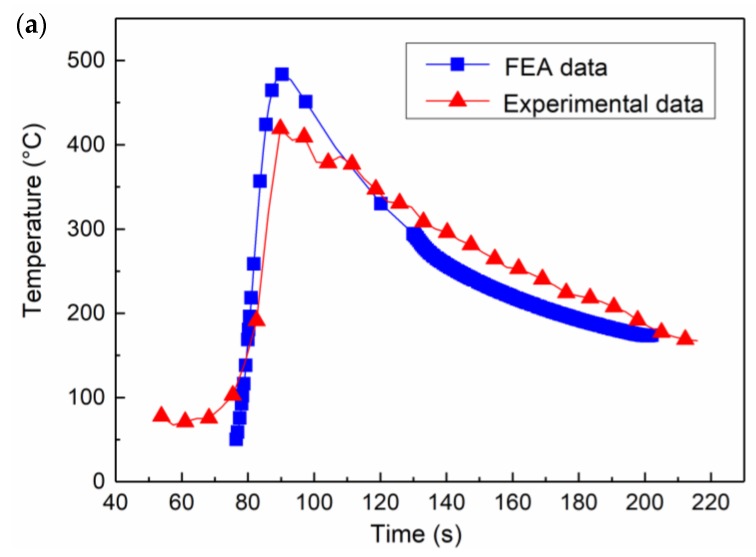

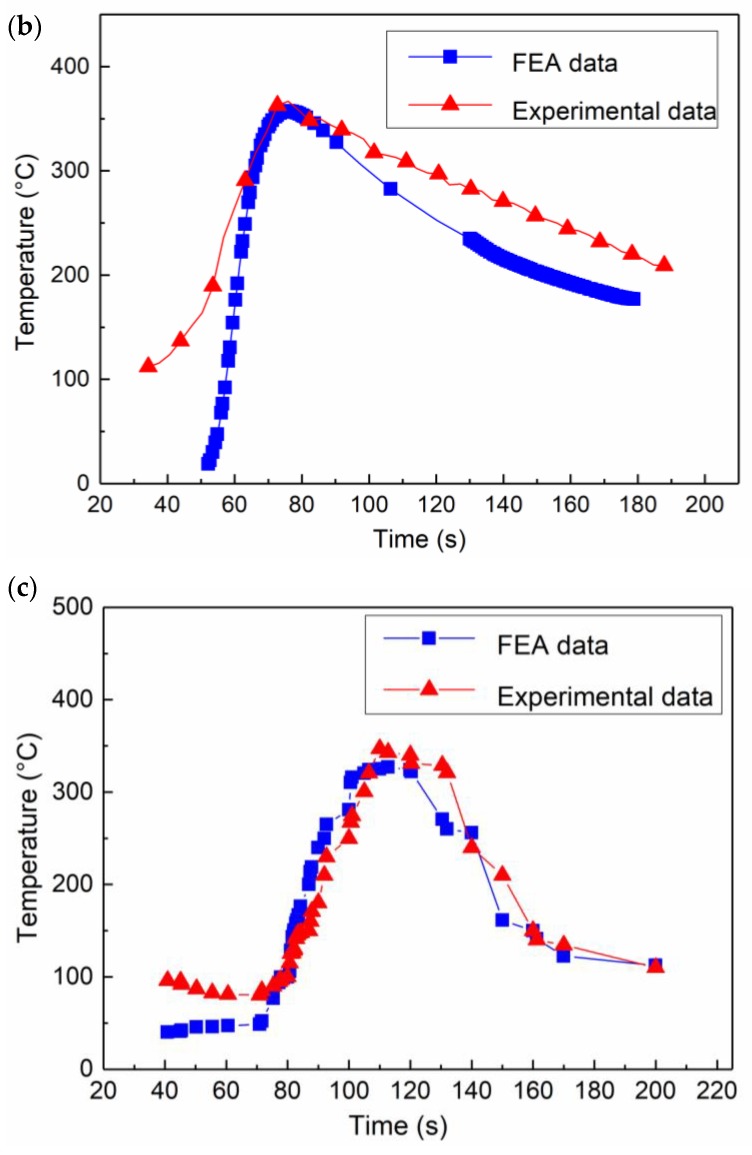
Comparison of the thermal cycle curves obtained from experiment and simulation at different points, *d* (**a**), *e* (**b**), and *f* (**c**). For the specific positions of *d*, *e*, and *f* points, refer to Figure 3.

To verify the accuracy of simulation of welding temperature distribution, the three corresponding points (*a*, *b*, and *c*, refer to Figure 3) in the welded composite plate were selected for experimental measurement by thermocouple method. The comparison of FEA and experimental results are shown in Figure 8. Through comparing the results from experiment and simulation, it can be seen that the FEA results are in good agreement with the experimental ones, which verifies the validity and accuracy of the model developed and provides an effective method for evaluating the distributions of residual stress and thermal stress in the welding of the composite plate.

### 3.2. Residual Stress Distribution

**Figure 9:**
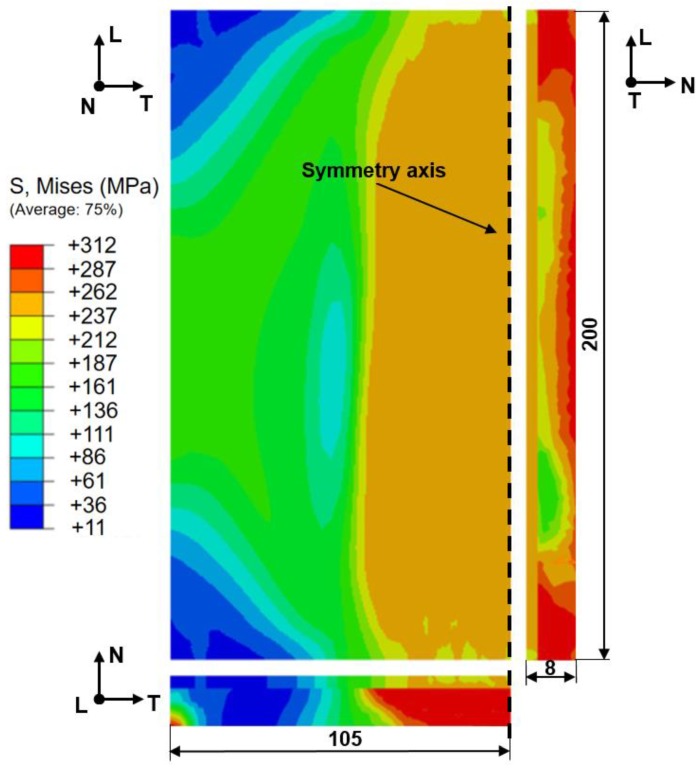
Von Mises residual stress distribution of the weld joint, which presents a half of the symmetrical weld joint.

Figure 9 shows the von Mises residual stress distribution of the weld joint. As can be seen from Figure 9, the larger residual stress of composite plates is distributed in the weld zone, and the maximum residual stress of the base metal reaches up to 312 MPa, which is higher than the maximum residual stress of 262 MPa in the clad metal layer. In addition, the maximum residual stress is mainly distributed at the starting and ending spots of the fusion line. Due to the difference in physical properties between the 304 SS and Q345R low alloy steel, the microstructure and mechanical properties are varied because of the occurrence of carbon migration near the fusion line. There is a stress discontinuity observed at the junction of the two materials.

**Figure 10:**
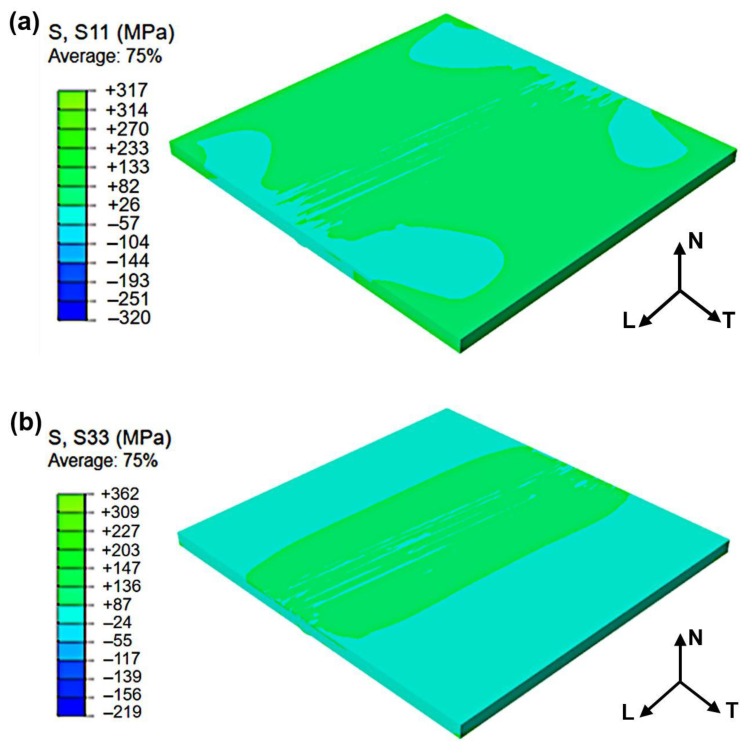
(**a**) Distributions of transverse stress component (S11) and (**b**) longitudinal stress component (S33) of the composite plate welding joint.

The residual stress components in *L*, *T*, and *N* direction are defined as the stresses in S11, S33, and S22 directions in FEA results, respectively. Figure 10 shows a transverse and longitudinal residual stress distribution of the composite plate. It can be seen from Figure 10a that the transverse stress of the composite plate is characterized by the simultaneous presence of tensile and compressive stresses. The most stress components in S11 direction distributed on the clad layer are tensile, and the maximum stress is 317 MPa. Figure 10b shows that the stress components in S33 direction distributed along with the fusion line are mainly tensile and the stress components which are far from the fusion line are compressive. The reason might be that during the heating process, the expansion of the weld causes extrusion on the starting and ending spots of the weld joint of base metal. The maximum stress component in S33 is 362 MPa in tension.

**Figure 11:**
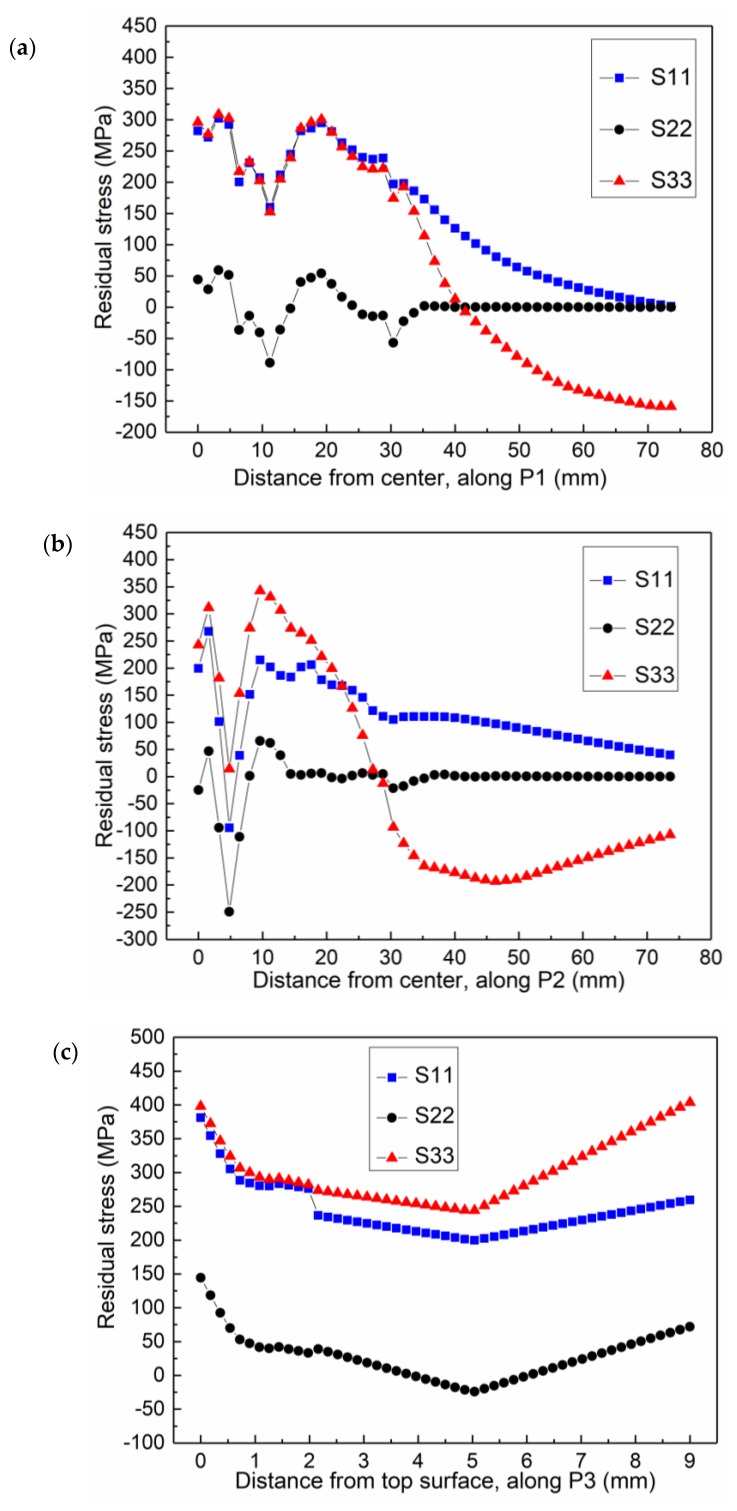
Residual stress distributions at different paths on the cross section of the composite welding joint: along P1 path (**a**), P2 path (**b**), and P3 path (**c**). For the specific positions of the paths, refer to Figure 1.

To find the relationship between the residual stress and the distance to the center of fusion line in the welding process of the composite plate, three paths (P1, P2, and P3) are selected as shown in Figure 1b for further analysis; the transverse stress S11, the normal stress S22, and the longitudinal stress S33 are analyzed, respectively. Figure 11a shows the distribution of residual stress of the clad metal in the path P1. It can be seen that the maximum residual stress is distributed in the weld zone. The fluctuation of the residual stress occurs at the junction connecting the fusion zone and HAZ. Since the clad layer needs to be welded six times, the weld is expanded by heating to cooling and contracted in each welding process. The form of residual stress alternates between tensile stress and compressive stress. In the clad layer, the transverse stress has large tensile stress with a maximum value of 276 MPa. The longitudinal stress is tensile stress with a maximum value of 283 MPa. The clad metal layer is manufactured by explosion welding and residual stress is generated in the composite plate. The residual stress is mainly distributed in the weld zone and HAZ. In the transition region from HAZ to matrix metal, the residual stress tends to be stable.

As shown in Figure 11b, along the P2 path, the stress in the weld zone gradually changes from tensile to compressive, and the magnitude of the stress gradually increases in the HAZ. The stress gradually decreases and tends to be stable after reaching the base metal zone. Since the clad layer weld is thermally expanded, a bending effect is generated on the base layer during the welding process and the bending is mainly caused by stress in the S11 direction. However, the lower surface of the base metal has a relatively lower temperature, which hinders the expansion of the upper surface. The stress in S11 compresses the center of the weld joint in the base metal to form compressive stresses. The stress along the thickness is mainly manifested as tensile stress in the weld zone and HAZ. During repair welding on the clad metal layer, the expansion caused by heat will press both sides of the base metal in the direction of thickness.

Figure 11c shows the residual stress distribution along the path P3. The stress distribution along the thickness direction is uneven. During the transition from the clad layer to base metal layer, the stress gradually decreases. However, at the junction of the first and second weld of the base metal layer, the stress starts to increase gradually from a specific point, which is 5 mm away from the starting point of the path P3. The FEA results extracted from corresponding points as illustrated in Figure 3 are compared with the results obtained by the blind-hole method to verify the validity of the numerical model. The comparison of residual stresses obtained from FEA and experiment is shown in Table 4. Table 4 shows that the relative errors of transverse stress at point *e* and longitudinal stress at point *f* are relatively larger, they are 14% and 18%, respectively. The other relative errors are all within 18.0%. Pearson correlation coefficient is also used to verify the validity and accuracy of the numerical model. The Pearson correlation coefficient, which was developed by Karl Pearson [14], is a measure of the linear correlation between two variables X and Y. According to the Cauchy–Schwarz inequality, it has a value between +1 and −1, where 1 is total positive linear correlation, 0 is no linear correlation, and −1 is total negative linear correlation. The equation of Pearson correlation coefficient is as follows:

(3) $$\rho_{\left(X,Y\right)}=\frac{\mathrm{cov}(X,Y)}{\sigma_{X}\sigma_{Y}}=\frac{E((X-\mu_{X})(Y-\mu_{Y}))}{\sigma_{X}\sigma_{Y}}=\frac{E(XY)-E(X)E(Y)}{\sqrt{E(X^{2})-E^{2}(X)}\cdot \sqrt{E(Y^{2})-E^{2}(Y)}},$$

where *E* is expectation and cov is covariance, $\sigma_{X}$ and $\sigma_{Y}$. are the standard deviations of *X* and *Y*, $\mu_{X}$ and $\mu_{Y}$ are the means of *X* and *Y*, respectively. Three transverse residual stress components obtained from the experiment and FEA are taken into Equation (3) as *X*_1_, *Y*_1_, represented by *X*_1_ < 193.90, −19.54, 44.89> and *Y*_1_ < 180.41, −30.01, 48.06>. The longitudinal residual stress components in Equation (3) are also taken. *X*_2_ and *Y*_2_ are the stress components obtained from the experiment and FEA, respectively; *X*_2_ < 147.77, −170.52, 3.43> and *Y*_2_ < 121.17, −140.32, 15.61>. *X*_1_, *Y*_1_, *X*_2_, and *Y*_2_ are substituted into Equation (3) and the results are $\rho_{S11(X_{1},Y_{1})}=0.99706$, and $\rho_{S33(X_{2},Y_{2})}=0.99838$. The above results are close to 1, indicating that the FEA results have a good linear correlation with the experimental ones. Therefore, it can be concluded that the results obtained from simulation and experiment are in good agreement, which proves that the finite element model in this study is able to accurately predict the welding residual stress distribution of the composite plate weld joint.

### 3.3. Microstructure Characterization and Evolution

**Figure 12:**
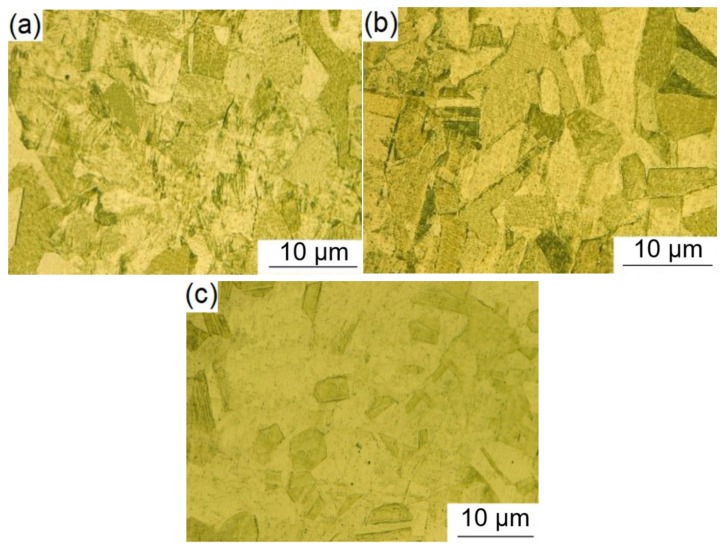
Microstructures of specimens sampled at points *g* (**a**), *h* (**b**), and *i* (**c**) (referring to Figure 4) in the weld joint of the composite plate.

The microstructures of the composite layers are shown in Figure 12a–c, corresponding to points *g*, *h*, and *i* as shown in Figure 4. The mean grain sizes of austenite in Figure 12a–c were 31.8 μm, 36.4 μm, and 40.2 μm, respectively, by means of intercept method [15]. The grain sizes increased successively. Austenitic grains in Figure 12a are uniform and small in size. Compared with Figure 12a, the austenitic grains in Figure 12b are relatively larger in size, while austenitic grains in Figure 12c are much larger. Since the sampling position in Figure 12a is close to the weld joint, it must be greatly affected by the welding heat. The austenitic grain is finer and more uniform. The sampling position in Figure 12c is far from the welded joint and relatively less affected by the welding heat. In the process of base layer welding, the heat will affect the microstructure of the weld groove.

**Figure 13:**
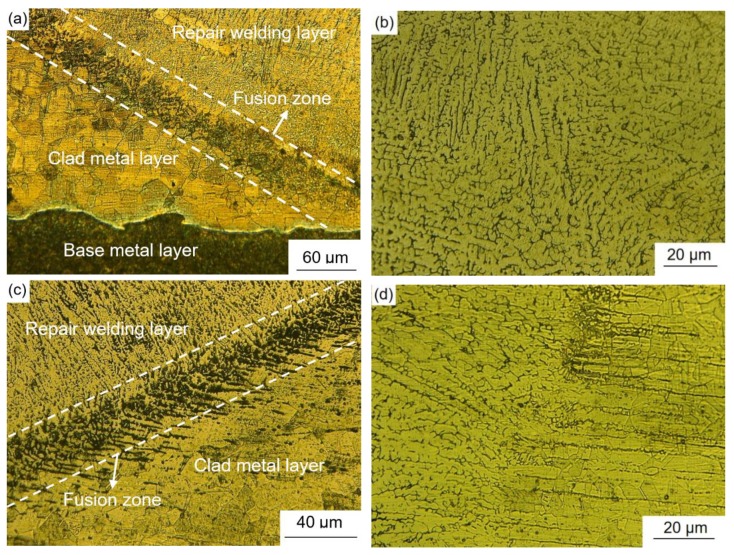
Microstructures of specimens sampled at points of *j* (**a**), *k* (**b**), *m* (**c**), and *l* (**d**) in the weld joint. For the specific positions of the points, refer to Figure 4.

Figure 13 shows the microstructures of the interfaces of the explosive weld of 304 SS/Q345R, and interfaces of repair welding and clad layer metal, marked as *j*, *k*, *l*, and *m* points as shown in Figure 4. The interfacial weld between the clad layer and the base layer is wavy [16,17]. Figure 13a shows the microstructures of the joint of two interfaces, including the interface of explosive weld of 304 SS/Q345R and interface of repair welding and clad layer metal. There is a fusion zone at the interface between the 304 SS and Q345R layers. The microstructure of the interface is shown in Figure 13a. The grains in the fusion zone and at both sides of the fusion zone are all austenite, but the austenite grain of the fusion zone is finer. Below the wavy boundary is the microstructure of Q345R base layer, which is mainly composed of ferrite and pearlite. Figure 13b shows the microstructure of the repair welding in clad metal. It can be seen that black vermicular ferrite is distributed in the austenite matrix [18]. The mechanism of the microstructure formation may be that during the solidification of the weld, the presence of *δ*-ferrite hinders the growth of columnar austenite grains. Due to the large heat input in the welding process of the composite layer, the content of ferrite is likely to be high. The presence of ferrite will improve the corrosion resistance of the welded joints to a certain extent and prevent the occurrence of hot cracks in the weld. Figure 13c shows the microstructure of interface between repair welding and clad layer. A fusion zone is formed between the weld and the composite layer, which is mainly composed of lath-like and needle-like ferrites. Near the fusion line, the microstructure of weld metal is columnar austenite grain and the grain is smaller than that observed in other regions. The austenite structure of the weld metal is usually attached to the surface of composite layer and begins to crystallize. As can be seen in Figure 13c, the inner weld forms columnar grains and the grains are refined. The reason may be that the outer weld melts the inner weld and begins to recrystallize on the surface. Due to the dilution effect of the composite layer, a large number of rare elements including Ni and Cr in the composite layer are diffused into the base layer [19], which reduced the content of Cr and Ni in the composite layer. As a result, the microstructure and composition of the welding seam and transition zone are significantly different, forming a relatively obvious boundary line. Figure 13d shows the microstructure of the welding seam and base metal of 304 SS. The austenite mainly presents a stripped shape. Since there is no significant difference between the composition and structure of the welding seam and the transition area, no obvious dividing line is observed between the welding seam and the transition zone.

**Figure 14:**
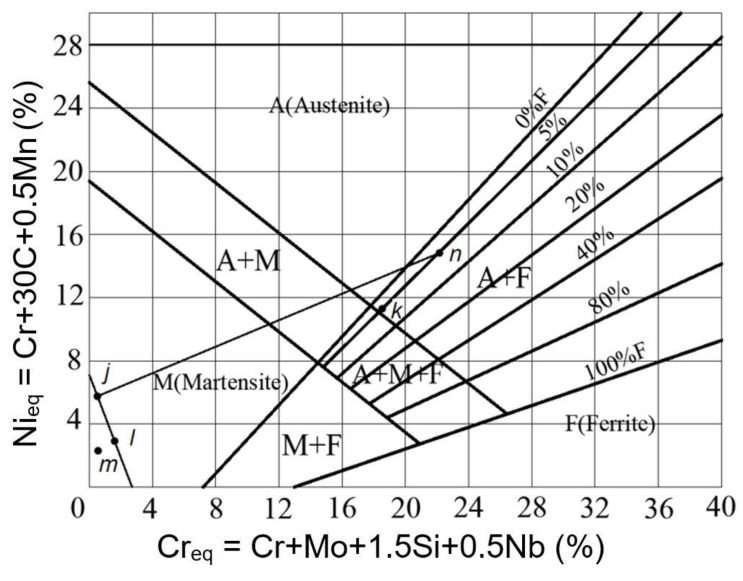
Schaeffler diagram.

The Schaeffler diagram, as shown in Figure 14, can be used to predict the proportion and phase composition of the weld microstructure. The axes of *x*, *y* in the coordinate of the diagram represent chromium (Cr) equivalent and nickel (Ni) equivalent, respectively. Schaeffler diagram is an important tool to predict Cr-Ni austenite, austenite-ferrite, or austenite-martensite weld. Table 5 shows the Cr equivalent and Ni equivalent values of the bimetal and filler rods. In the Schaeffler diagram, *j* and *k* points, corresponding to Q345R and 304 SS, present the equivalent values of Ni and Cr of the bimetals. While the filler rods of Er50-6, J507, and 302 SS correspond to *l*, *m*, and *n* points, respectively. It can be seen that both the filler materials of 302 SS and plate 304 SS fall in the two-phase zone of austenite and ferrite. The Ni and Cr equivalent of 302 SS is relatively higher, of which phase composition points fall in the zone with 5–10% ferrite content [20]. The Ni and Cr equivalent of 304 SS is relatively smaller, of which phase composition points fall in the zone with 10–20% ferrite content. The amount of Ni and Cr in the composite layer is small, of which ferrite content is about 5% and the phase components fall at the edges of the austenite and ferrite three-phase regions. Studies have shown that a certain amount of ferrite can promote the formation of an ideal ferrite/austenite interface, and the presence of 5–10% ferrite can effectively improve the hot crack resistance and resistance to intergranular corrosion of composite welded joints [21]. Due to the high hardness of ferrite, the proper amount of ferrite has a positive effect on improving mechanical properties of the composite plates. Since ferrite will form σ, which is a brittle and hard phase at high temperatures, the σ phase will reduce the corrosion resistance and strength properties of the composite plate. Hence, ferrite content cannot be too high for the components, which are in a long-term high temperature operating environment.

**Figure 15:**
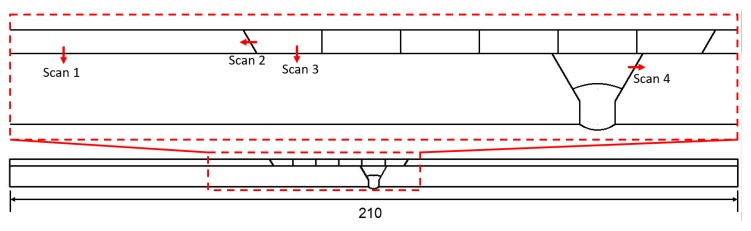
Positions of energy-dispersive X-ray spectroscopy line scans performed on the cross section of the composite plate weld joint.

**Figure 16:**
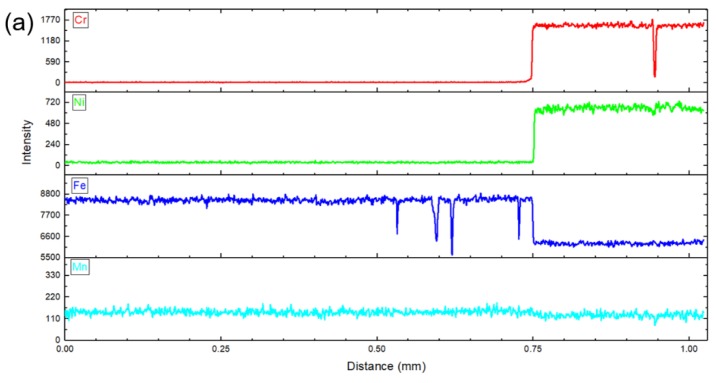

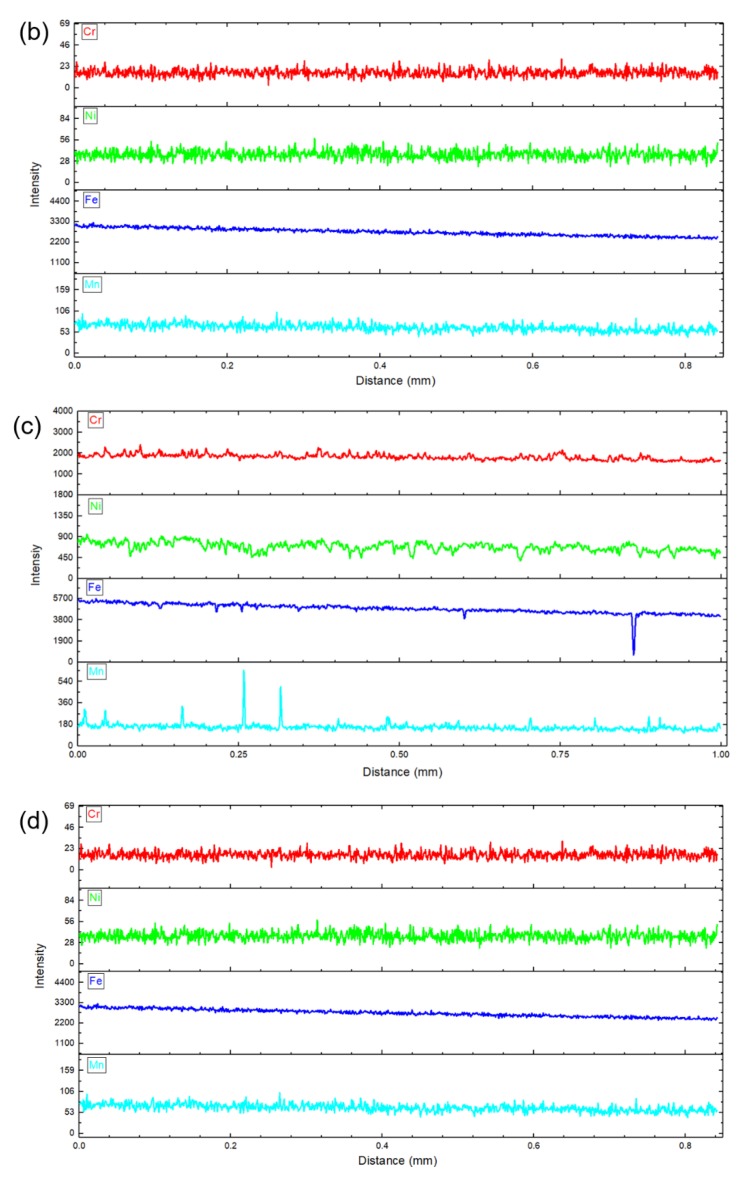
The element intensities of Cr, Ni, Fe, and Mn along the line-scan paths Scan 1 (**a**), Scan 2 (**b**), Scan 3 (**c**), and Scan 4 (**d**).

The line scans of EDS on the weld joints of the 304 SS/Q345R composite plate are carried out to analyze the migration and diffusion of elements between the base metal and the weld zone. Figure 15 shows the four scanning paths, namely, Scans 1, 2, 3, and 4. Intensity (or counts) is the number of photoelectrons measured, and its signal strength can reflect the changing trend of element content qualitatively. It can be seen from Figure 16a that the signal intensities of elements of Ni, Cr, Fe, and Mn have a sudden change at the interface between the base metal and clad layers due to the different chemical compositions of 304 SS and Q345R low alloy steel. In addition, the sudden change in chemical composition is also due to the fact that the explosive welding is completed in an instant, without excessive heat affecting the further diffusion of the elements. Scan 2 was crossing the interface of the repair welding and clad layer, as shown in Figure 16b. A slight decrease was observed in the elements of Cr, Ni, Fe, and Mn along the scan path, because there are only slight differences between the chemical compositions of 304 SS and 304 SS. Figure 16c shows the interface between 302 SS filler material and base metal, compared with the result in Figure 16a, there is no obvious change along the path, the reason is mainly due to significant element diffusion caused by the welding heat from several repair weldings. Figure 16d shows the results of Scan 4 crossing the interface between the base metal layer and the second welding for the base layer. From the start point to the end of the Scan 4 path, the elements of Cr and Ni remain unchanged along the scan path, while the elements of Fe and Mn tend to reduce a little due to the differences for the two elements in Q345R and filler material J507.

## 4. Conclusions

In this study, the 304 SS/Q345R composite plate welding and repair welding joint were prepared. The thermomechanical behaviors, including residual stress distribution and thermal cycle curves, were studied based on the thermos-elastic-plastic theory by means of experimental and numerical methods. The evolution of the microstructure and residual stress distributions of the composite weld joint were also investigated. The influence of the welding temperature evolution on the microstructure and mechanical properties of the welding joint were investigated. The main conclusions can be drawn as follows:

1. During the welding process, the influence of the heat source on the measured point will gradually decrease with the increase in distance between the welding joint and the measured point, and the maximum temperature is 2140 °C in the third welding process. FEA results are in good agreement with the experimental ones, which verifies the validity and accuracy of the model developed and provides a practical method for evaluating the distributions of temperature and residual stress.
2. The FEA results show that the maximum transverse tensile stress of the joint is mainly distributed near the fusion line. The maximum longitudinal tensile stress appears in the weld zone and the HAZ, and its value is 283 MPa. During the transition from weld and HAZ to base metal zone, the stress gradually decreases from the maximum 312 MPa to 0 and tends to be stable. During the welding process, the residual stress produces bending deformation to the base plate due to te repeated welding. The simulation results of transverse stress and longitudinal stress are in good agreement with the results obtained by the blind-hole method, which proves that the finite element model developed in this study can accurately predict the residual stress distribution of the weld joint of composite plates.
3. The microstructure of the 304 SS/Q345R composite plate welded joint is mainly composed of austenite and ferrite. The ferrite near the fusion line forms a transition region in the shape of a strip, while the austenite near the weld line is mainly composed of columnar grains. The mean grain size is smaller than those in the base metal.
4. A phenomenon of residual stress discontinuities is observed at the weld interface, which is worth further study.

## Figures and Tables

**Table 1:** The chemical composition of 304 SS and Q345R low alloy steel (wt.%).

Material	C	Si	Mn	P	S	Ni	Cr	Fe
304 SS	0.07	0.345	1.091	≤0.045	0.036	8.215	18.09	Balance
Q345R	0.2	0.247	0.604	≤0.025	0.04	0.061	0.052	Balance

**Table 2:** The chemical composition of the filler rods (wt.%).

Filler Rod	C	Mn	Si	S	P	Ni	Cr	Mo	Cu	Fe
Er50-6	0.105	0.163	0.975	0.013	0.015	0.016	–	0.06	–	Balance
J507	0.12	1.60	0.75	0.035	0.040	0.30	0.20	0.30	–	Balance
302 SS	0.064	0.80	0.70	0.010	0.030	12.50	24.00	0.40	0.20	Balance

**Table 3:** Technological parameters of the welding process.

Welding Sequence	Current (A)	Voltage (V)	Welding Time (s)	Cooling Time (s)	Filler Rod Type	Filler Rod Diameter (mm)
1	122	20	125	154	J507	3.2
2	122	20	64	57	J507	3.2
3	122	20	32	~0	302 SS	3.2
4	122	20	35	~0	302 SS	3.2
5	122	20	33	~0	302 SS	3.2
6	122	20	35	~0	302 SS	3.2
7	122	20	35	~0	302 SS	3.2
8	122	20	50	Cool to room temperature	302 SS	3.2

**Table 4:** The comparison of residual stresses obtained from FEA and experiment.

Measurement Points	Stress Component	Experimental (MPa)	FEA (MPa)	Relative Error (%)
Point *d*	Transverse stress	193.90	180.41	7
	Longitudinal stress	147.77	121.17	18
Point *e*	Transverse stress	−19.54	−22.33	14
	Longitudinal stress	−170.52	−161.47	5
Point *f*	Transverse stress	44.89	48.06	7
	Longitudinal stress	3.43	4.05	18

**Table 5:** The equivalent quantities of Cr and Ni in the bimetal and filler rods (wt.%).

Element	304 SS	Q345R	Er50-6	J507	302 SS
Cr_eq_	18.6	0.4	1.5	1.2	22.3
Ni_eq_	10.9	6.1	3.2	2.8	14.8
Cr_eq_/Ni_eq_	1.7	0.1	0.5	0.4	1.5
